# Efficacy and safety of CD38-directed CAR-T cell therapy for multiple myeloma: a systematic review and meta-analysis

**DOI:** 10.3389/fonc.2026.1744250

**Published:** 2026-04-29

**Authors:** Xinlong Xu, Chang Dong, Jiashuo Guo, Xiaolin Chang, Yu Zhang, Shuting Gou, Liying Xue, Jie Li

**Affiliations:** 1 Graduate School, Hebei North University, Zhangjiakou, China; 2 Department of Hematology, Hebei General Hospital, Shijiazhuang, China; 3 Department of Pathology, Hebei Medical University, Shijiazhuang, China

**Keywords:** CD38, chimeric antigen receptor T-cell therapy, efficacy, meta-analysis, multiple myeloma, safety

## Abstract

**Background:**

Relapsed/refractory multiple myeloma (RRMM) remains a clinical challenge despite therapeutic advances. CD38-directed chimeric antigen receptor T-cell (CAR-T) therapy, especially dual-target CD38/BCMA constructs, represents an emerging immunotherapeutic strategy. This systematic review and meta-analysis aimed to evaluate the efficacy and safety of CD38-directed CAR-T in RRMM.

**Methods:**

We systematically searched Medline, Embase, and Cochrane Library up to October 1, 2025. Eligible clinical trials investigating CD38-directed CAR-T (single-target or dual-target CD38/BCMA) for RRMM were included. Random-effects model was used to pool efficacy and safety outcomes of dual-target studies, while single-target CD38 data were summarized descriptively.

**Results:**

A total of 4 studies involving 70 patients were included (3 dual-target CD38/BCMA studies, n=61; 1 single-target CD38 study, n=9). For dual-target CD38/BCMA CAR-T, the pooled overall response rate (ORR) was 89% (95% CI: 81%–97%), complete response/stringent complete response (CR/sCR) rate was 63% (95% CI: 44%–82%), and minimal residual disease (MRD)-negative rate was 67%. Mortality was 11%. The single-target CD38 CAR-T showed lower efficacy (ORR = 33%) and higher mortality (44%). Any-grade cytokine release syndrome (CRS) occurred in 83% of patients, with grade ≥3 CRS in 26%. Other adverse events included infections (23%), immune effector cell-associated neurotoxicity syndrome (ICANS, 13%), and kidney injury (13%).

**Conclusion:**

Dual-target CD38/BCMA CAR-T demonstrates promising efficacy and manageable safety in RRMM. Evidence for single-target CD38 CAR-T remains limited and requires cautious interpretation. Further large-scale comparative studies are warranted to determine the optimal role of CD38-directed CAR-T in RRMM treatment sequencing.

## 1 Introduction

Multiple myeloma (MM) is the second most common hematologic malignancy, characterized by clonal proliferation of malignant plasma cells in the bone marrow and profound immune dysregulation that facilitates immune evasion and disease progression (1, 2). Despite substantial therapeutic advances, including proteasome inhibitors, immunomodulatory drugs, monoclonal antibodies, and autologous stem-cell transplantation, the prognosis of patients with relapsed/refractory multiple myeloma (RRMM) remains unsatisfactory. Tumor- and stroma-derived cytokines, including TNF and IL-6, promote immune dysfunction and support myeloma growth, influencing responses to immunotherapy (3, 4). The 5-year overall survival has improved from 30% to 60% in recent decades (5–8), yet MM-related mortality remains high (9). Patients with high-risk features, such as adverse cytogenetics or extramedullary disease, show particularly poor outcomes (1, 10, 11). Most patients eventually relapse and become refractory to standard treatments, highlighting an urgent need for innovative therapeutic strategies.

Chimeric antigen receptor T−cell (CAR−T) therapy has emerged as a transformative immunotherapeutic approach for RRMM (12–14). Multiple targets have been investigated, including B−cell maturation antigen (BCMA), CD19, CS1, CD38, GPRC5D, κ light chain, and NKG2D ligands (15–31). BCMA−targeted CAR−T has demonstrated remarkable efficacy, yet antigen escape and disease recurrence remain common, supporting the development of alternative or combinatorial targets. CD38 is a type−II transmembrane glycoprotein universally overexpressed on malignant plasma cells in MM, with low expression on normal lymphocytes and non−hematopoietic tissues, thus offering a favorable therapeutic window (32, 33). CD38−directed CAR−T cells, especially dual−target CD38/BCMA constructs, have been evaluated in early−phase clinical trials (26–28); however, a systematic assessment of efficacy and safety is still lacking.

Bispecific antibodies and CAR−T have reshaped the RRMM treatment landscape, but their toxicity profiles and optimal positioning require further clarification (34). To date, no systematic review and meta−analysis has quantitatively synthesized clinical evidence of CD38−directed CAR−T therapy in RRMM. Therefore, we performed this systematic review and meta−analysis to evaluate the efficacy and safety of CD38−directed CAR−T cell therapy in patients with RRMM, with separate analyzes for dual−target CD38/BCMA and single−target CD38 constructs. This study aims to provide evidence−based guidance for clinical practice and future trial design.

## 2 Methods

### 2.1 Search strategy

This study was performed according to PRISMA (Preferred Reporting Items for Systematic Reviews and Meta-Analyses). We conducted a systematic search of the Medline, Embase, and the Cochrane Library databases for cohort studies on CD38 CAR-T cell therapy for multiple myeloma, with a search period covering the databases inception to October 1, 2025. Additionally, we traced the references of included studies to supplement literature acquisition. The search strategy combined Medical Subject Headings and free-text terms related to multiple myeloma and CAR-T cell therapy. The main search terms included: (“multiple myeloma” OR “MM”) AND (“chimeric antigen receptor T cell” OR “CAR-T” OR “CAR T”) AND (“CD38” OR “CD38 CAR-T”).

### 2.2 Inclusion and exclusion criteria

Two scholars independently screened the literature, extracted the data and checked them. If they did not agree, they discussed or consulted a third party to solve the problem. The research without data was not included. The following criteria were required for inclusion: (1) Original studies involving eligible multiple myeloma patients, (2) Therapies using CD38 CAR-T either as monotherapy or in combination with other CAR-T therapies (e.g., BCMA), (3) At least one primary endpoint: Overall Response Rate (ORR), Stringent Complete Response (sCR), Complete Response (CR), Very Good Partial Response (VGPR), Partial Response (PR), or mortality. (4) The study must be a clinical trial and published in English, (5) Non-repeated publications or data, (6) Studies with more than three enrolled patients.

### 2.3 Quality assessment and data extraction

The study investigates the problem through three dimensions: target population, intervention measures, and outcome patterns. The research focuses on multiple myeloma patients and CD38 CAR-T cell therapy. The outcomes include post-treatment prognosis and adverse reactions. Key data extracted included: first author, publication year, study design/phase, sample size, patient demographics (age, sex, ethnicity), and disease characteristics (staging and cytogenetics when available). We additionally extracted treatment-related variables whenever reported, including the number of prior lines of therapy, prior exposure to major drug classes (e.g., proteasome inhibitors, immunomodulatory drugs, and anti-CD38 antibodies), combination/bridging regimens, CAR-T product features (target(s), construct, and dose), lymphodepletion, and follow-up duration. Efficacy endpoints included: ORR, sCR, CR, VGPR, PR, Minimal Residual Disease (MRD) negativity, and mortality. Safety endpoints encompassed adverse reactions such as cytokine release syndrome (CRS) (by grade), hematologic toxicity, infection, liver/kidney injury, gastrointestinal reactions, fever, and Immune-effector-Cell–Associated Neurotoxicity Syndrome (ICANS).

### 2.4 Statistical analysis and data synthesis

A random−effects model was applied to pool efficacy and safety outcomes of CD38−directed CAR−T therapy. Given construct heterogeneity, quantitative synthesis was focused on dual−target CD38/BCMA studies, while the single−target CD38 study was summarized descriptively. Proportions were pooled using the logit transformation and back−transformed for reporting. Between−study heterogeneity was assessed using the Q test and I² statistic, with significant heterogeneity defined as P < 0.10 or I² > 50%. Meta−regression and publication bias analyzes were not performed owing to the limited number of included studies. All statistical analyzes were conducted using Stata 14.0 software. The risk of bias was evaluated with the Newcastle−Ottawa Scale (NOS).

## 3 Results

### 3.1 Study selection and patients

**Figure 1:**
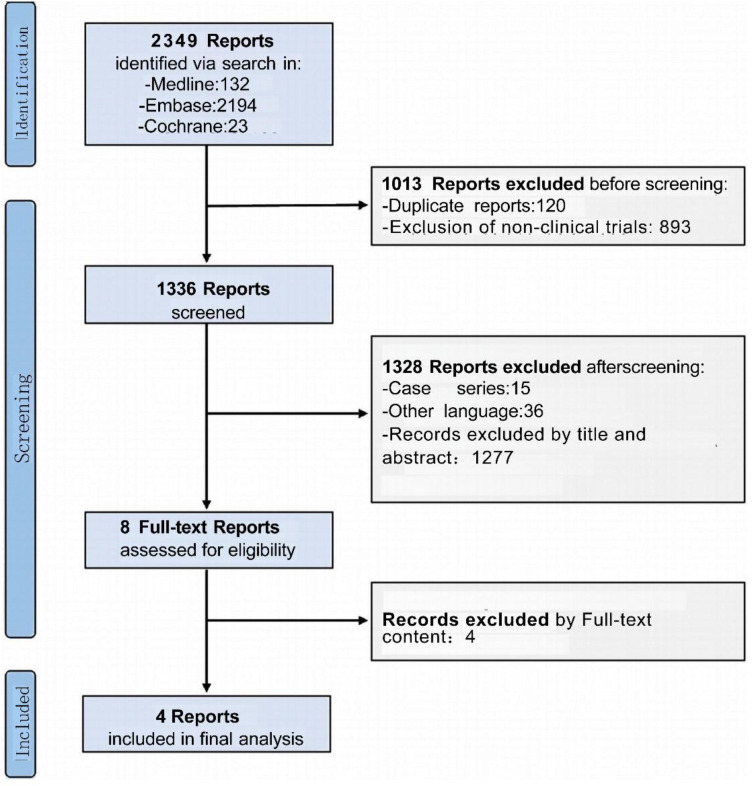
Flowchart for literature screening.

According to our search strategy, a total of 2349 articles were identified. After excluding duplicate articles, review articles, animal experiments, and irrelevant resources, 8 potential studies were screened for further evaluation. Following the exclusion of 4 studies that did not meet the eligibility criteria, four studies were ultimately included that met the inclusion criteria, involving a total of 70 patients (35–38). Three studies investigated dual-target CD38/BCMA CAR-T (n=61), and one study evaluated single-target CD38 CAR-T (n=9). The research screening flowchart is shown in **Figure 1**. A random-effects model was used in all pooled analyzes to accommodate inter-study heterogeneity.

All included studies were published between 2021 and 2023, comprising three phase I single-arm trials and one phase II trial (**Table 1**). The baseline characteristics showed that all enrolled patients were diagnosed with RRMM. The proportion of male patients was 47.8%, 40.9%, and 77.8% in three studies, respectively, while sex distribution was not reported in one study. Two studies adopted the R-ISS or ISS staging system, with a small proportion of patients at stage I. According to the Newcastle–Ottawa Scale (NOS), all 4 studies scored ≥7 points, indicating high methodological quality.

**Table 1:** Basic characteristics of the included studies.

Study ID	N	Sex (male %)	Median age	Prior proteasome inhibitor (N)	ECOG (0-2)	Stage (N)	Targets	Efficacy (N)	Death(N)	NOS
Mei et.al. 2021 (35)	23	47.8%	59	23	19	I:6, II:6, III:11 (RISS)	CD38+BCMA	ORR:20, CR/sCR:12, VGPR:4, PR:4, MRD:20	2	7
Zhang et.al. 2022 (36)	22	40.9%	56	22	9	I:0, II:17, III:5, (ISS)	CD38+BCMA	ORR:20, CR/sCR:12, VGPR:5, PR:3, MRD:10	2	8
Stadtmauer et.al. 2023 (37)	9	77.8%	61.6	9	9	I:3, II:2, III:2, unkown:1 (RISS)	CD38	ORR:3, CR/sCR:0, VGPR:0, PR:3	4	7
Tang et.al. 2022 (38)	16	–	58.5	16	–	I:2, II:1, III:12 unkown:1 (ISS)	CD38+BCMA	ORR:14, CR/sCR:13, VGPR:0, PR:1	4	7

indicates data not available or not reported in the original study.

### 3.2 Efficacy analysis

**Figure 2:**
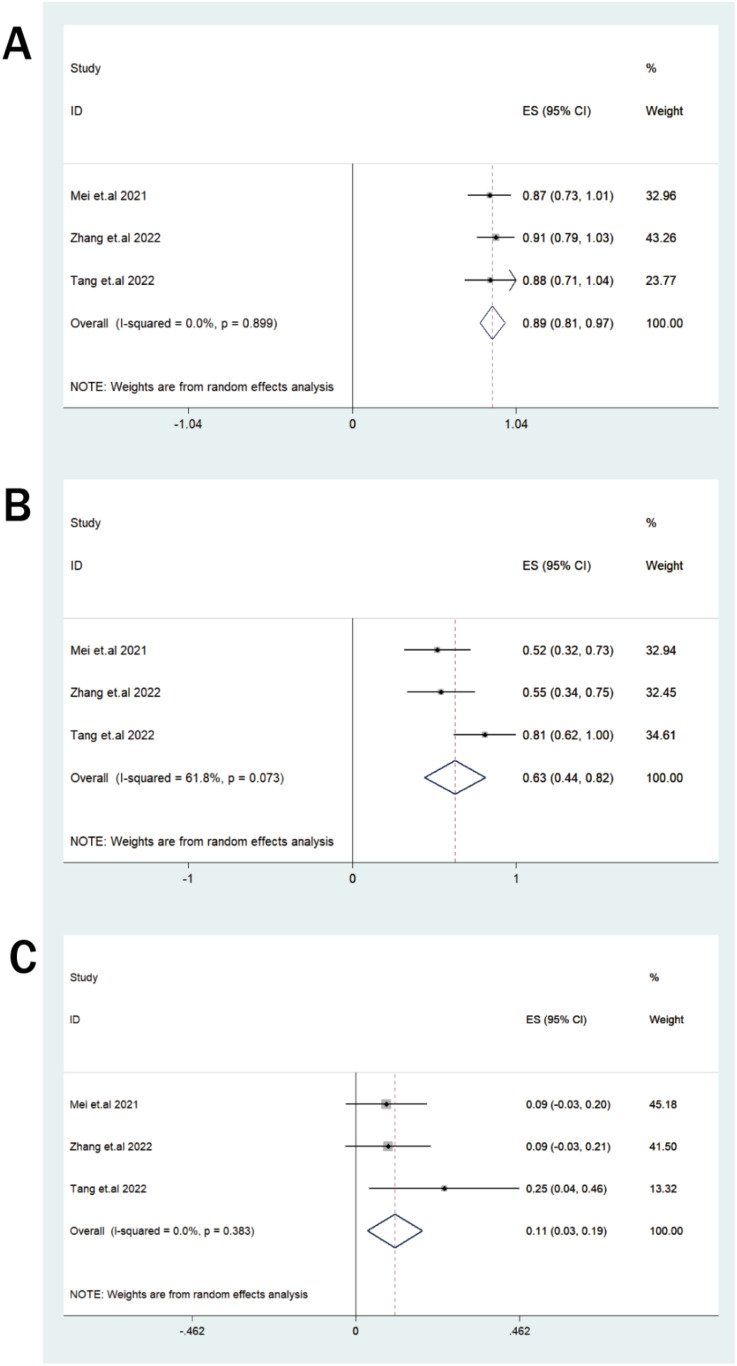
**(A–C)** Forest plots for efficacy outcomes in patients with RRMM receiving CD38-directed CAR-T cell therapy: **(A)** ORR, **(B)** CR/sCR, **(C)** mortality.

Four studies provided data for the efficacy meta-analysis. Given the construct heterogeneity, data from the single−target CD38 study were summarized descriptively, while the three dual−target CD38/BCMA studies were used for quantitative pooling. In the dual−target pooled analysis, the ORR was 89% (95% CI: 81%–97%; I²=0.0%, **Figure 2**). The pooled CR/sCR rate was 63% (95% CI: 44%–82%; I²=61.8%). Among two studies reporting MRD status, the pooled MRD−negative rate was 67% (95% CI: 26%–108%; I²=90.6%, **Supplementary Figure S1**). The pooled mortality was 11% (95% CI: 3%–19%; I²=0.0%). All key efficacy outcomes are listed in **Table 2** and **Figure 2**.

**Table 2:** Primary pooled efficacy and safety outcomes in the dual-target CD38/BCMA subgroup.

Outcome	Articles number	Heterogeneity (I²)	Heterogeneity (P value)	Response rate (95% CI)
ORR	3	0.0%	0.899	89% (81%–97%)
CR/sCR	3	61.8%	0.073	63% (44%–82%)
VGPR	2	0.0%	0.657	20% (8%–31%)
PR	3	0.0%	0.504	11% (4%–19%)
MRD negativity	2	90.6%	0.001	67% (26%–108%)
Mortality	3	0.0%	0.383	11% (3%–19%)
CRS (any grade)	3	0.0%	0.352	83% (72%–95%)
CRS (grade 1–2)	3	42.6%	0.175	62% (46%–78%)
Leukopenia	3	100.0%	NA	87% (73%–101%)
Anemia	3	80.5%	0.006	55% (29%–82%)
Thrombocytopenia	3	81.3%	0.005	38% (12%–65%)
Hematologic toxicity	3	100.0%	NA	96% (87%–104%)
Fever	3	48.3%	0.164	75% (55%–94%)
Infection	3	0.0%	0.407	23% (13%–34%)
Impaired liver function	2	24.9%	0.249	35% (19%–51%)
CRS (grade ≥3)	3	0.0%	0.788	26% (15%–37%)
Gastrointestinal reactions	3	60.4%	0.080	26% (9%–43%)
ICANS	2	0.0%	0.953	13% (3%–23%)
Kidney injury	2	0.0%	0.953	13% (3%–23%)

NA indicates not applicable due to extremely high between-study heterogeneity.

In the single−target CD38 study, the ORR was only 33% (3/9), with a PR rate of 33% (3/9) and 0% CR/sCR and VGPR. The mortality rate was as high as 44% (4/9) (**Table 1**).

### 3.3 Safety outcomes

**Figure 3:**
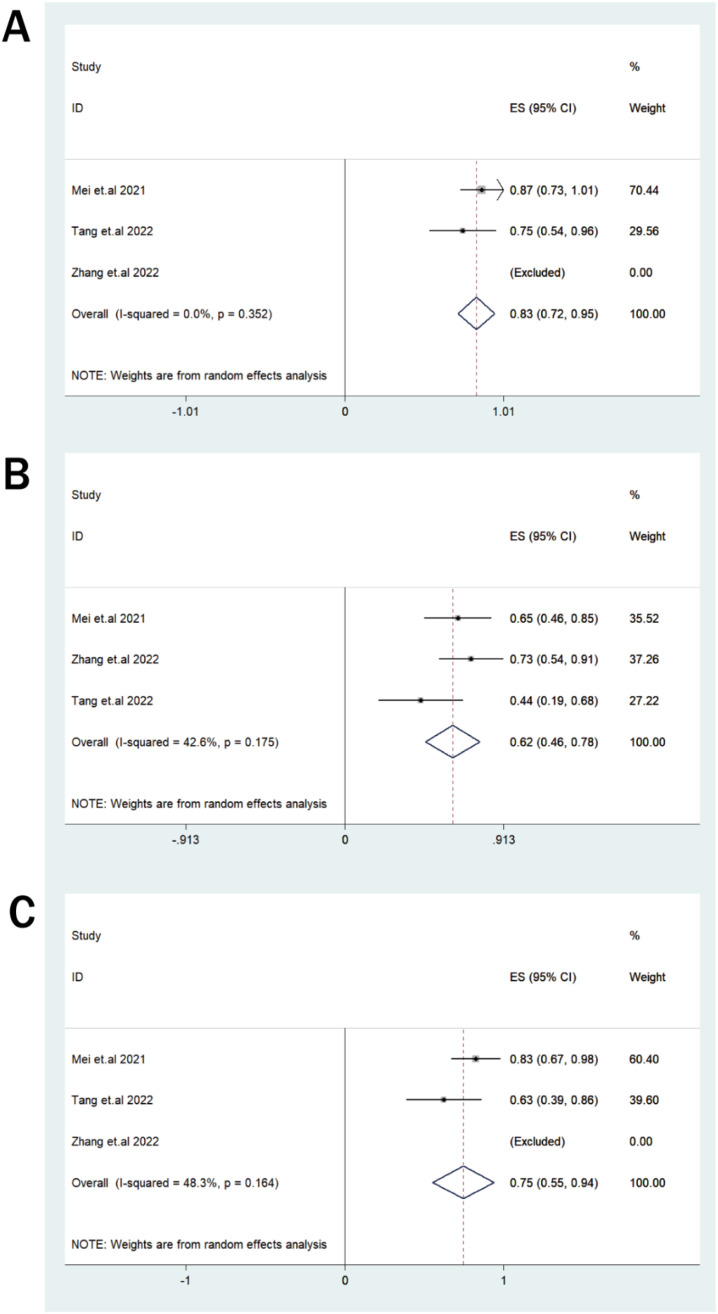
**(A–C)** Forest plots for common adverse events in patients with RRMM receiving CD38-directed CAR-T cell therapy: **(A)** CRS (any grade), **(B)** CRS(grade 1–2), **(C)** fever.

**Figure 4:**
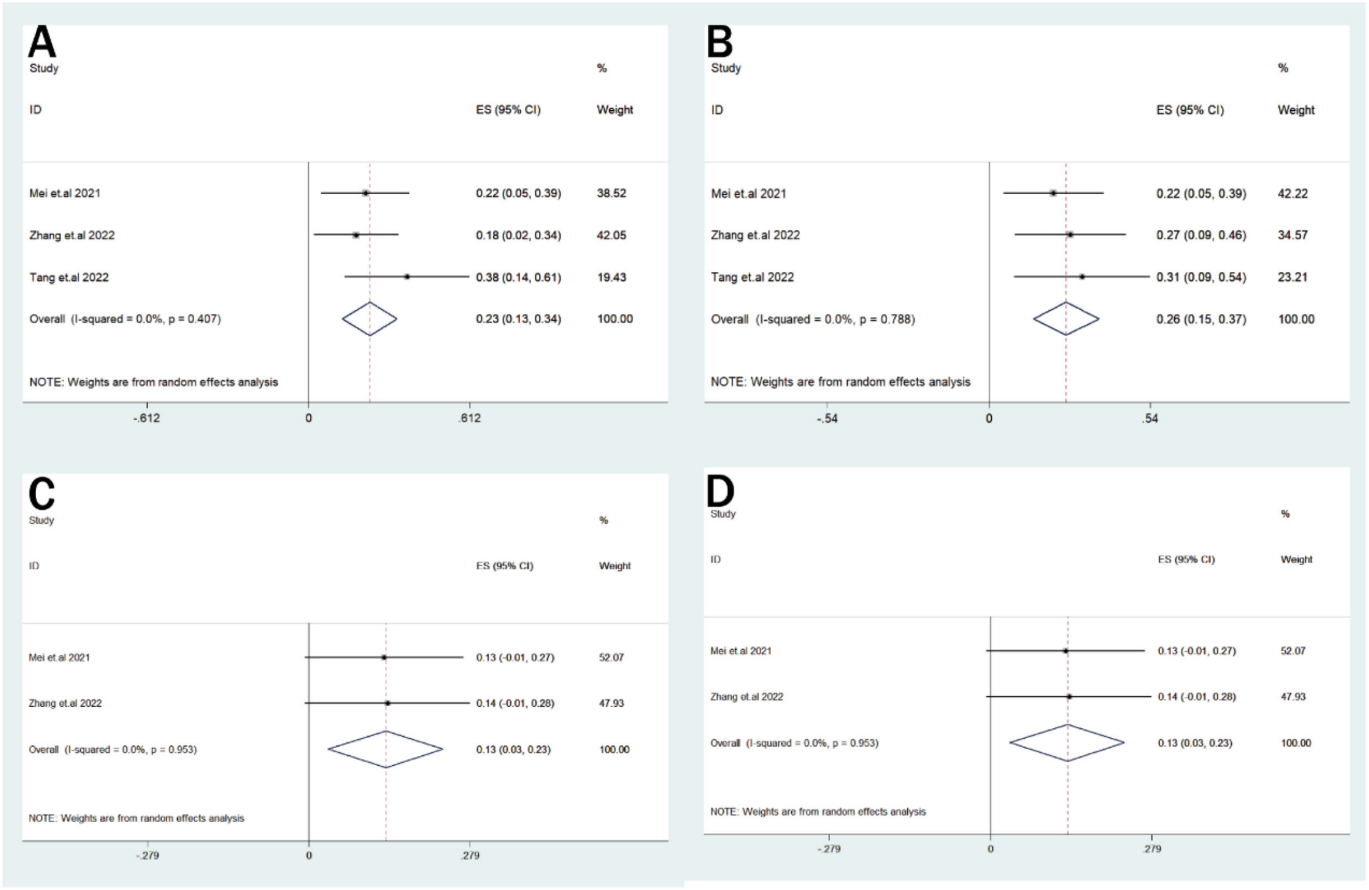
**(A–D)** Forest plots for other adverse events in patients with MM receiving CD38-directed CAR-T cell therapy: **(A)** infection, **(B)** CRS(grade ≥3), **(C)** kidney injury, **(D)** ICANS.

Safety outcomes were primarily pooled for the dual-target CD38/BCMA subgroup, whereas data from the single-target CD38 study were described narratively. In the dual-target pooled analysis, any-grade CRS occurred in 83% of patients (95% CI: 72%–95%; I²=0.0%), and grade 1–2 CRS occurred in 62% (95% CI: 46%–78%; I²=42.6%). Fever was reported in 75% of patients (95% CI: 55%–94%; I²=48.3%, **Figure 3**). The main safety results are summarized in **Table 2** and **Figure 4**.

Other notable adverse events included grade ≥3 CRS in 26% (95% CI: 15%–37%; I²=0.0%), infections in 23% (95% CI: 13%–34%; I²= 0.0%), ICANS in 13% (95% CI: 3%–23%; I²=0.0%), and kidney injury in 13% (95% CI: 3%–23%; I²=0.0%). Adverse event outcomes with substantial heterogeneity are provided in **Supplementary Figure 2** and **S3**.

## 4 Discussion

Despite remarkable advances in proteasome inhibitors, immunomodulatory agents, and anti−CD38 monoclonal antibodies (35), relapse and drug resistance remain major obstacles in multiple myeloma treatment (17). Patients with RRMM continue to have dismal outcomes, underscoring the urgent need for novel and effective therapeutic approaches.

This systematic review and meta-analysis confirmed the promising efficacy of CD38-directed CAR-T in RRMM. The pooled ORR reached 89%, with CR/sCR rate of 63%. Deep responses such as CR/sCR are rarely achieved with conventional therapies (39), supporting the superior activity of CAR-T in this population. Notably, 67% of patients achieved MRD negativity, a well-established surrogate for prolonged survival in multiple myeloma (40, 41), suggesting that CD38-directed CAR-T may induce durable remissions. The pooled mortality was 11%, with most deaths attributed to infection or circulatory failure; only one case was related to severe CRS (35–38).

Safety data showed that CRS was the most common adverse event. Any-grade CRS occurred in 83% of patients, with grade ≥3 CRS in 26%, indicating that most CRS events were mild to moderate and manageable. Hematologic toxicities were also frequent, including leukopenia (87%), anemia (55%), and thrombocytopenia (38%), which may be related to lymphodepletion regimens and potential effects of CD38-targeted CAR on hematopoietic progenitor cells (35). Other notable adverse events included fever (75%), infections (23%), liver dysfunction (35%), gastrointestinal reactions (26%), ICANS (13%), and kidney injury (13%). These findings highlight the necessity of careful monitoring and supportive care during treatment.

Mechanistically, resistance to CD38−directed therapy may occur via antigen modulation and immune escape (42–44). Previous exposure to anti−CD38 antibodies can downregulate surface CD38 expression through internalization and intercellular transfer, potentially reducing the efficacy of subsequent CD38−targeted CAR−T therapy (45–48). In addition, inflammatory cytokine dynamics may affect CAR−T expansion, persistence, and exhaustion, thereby influencing clinical response and relapse patterns (49).

In the context of available therapies for RRMM, CD38-directed CAR-T strategies should be interpreted alongside both standard anti-CD38 monoclonal antibody therapy (e.g., daratumumab) and the current BCMA-targeted CAR-T landscape (18, 21, 50, 51). While our pooled results suggest that CD38/BCMA dual-target constructs can achieve deep responses, any comparison with BCMA CAR-T products should be considered an indirect, cross-trial assessment because of differences in patient populations, prior therapies, CAR-T constructs/dosing, and follow-up duration across studies (35, 38, 52, 53). Therefore, head-to-head trials or well-designed matched-cohort studies are warranted to define the relative efficacy, durability, and safety of CD38-directed CAR-T cell therapy and to clarify its optimal positioning in treatment sequencing (52–54).

Long-term outcomes of CD38-directed CAR-T therapy remain poorly defined in the current literature (54–56). The included studies had relatively short follow-up, with inconsistent reporting of PFS and OS, which limits robust conclusions on response durability (35, 38, 57). In addition, late toxicities (e.g., prolonged cytopenias, late infections, delayed neurotoxicity) and relapse patterns related to antigen escape or target downregulation were not systematically collected (35, 58–61).

Our study has several limitations that should be acknowledged. First, the number of included studies was small and most were early-phase single-arm trials, restricting the precision of pooled estimates. Second, key baseline features, treatment variables, and high-risk stratification factors were inconsistently reported, limiting subgroup analyzes and meta-regression. Third, the definition and grading of adverse events varied across studies, further hampering comprehensive safety evaluation. Fourth, our search was limited to three major databases and English-language publications, and publication bias could not be formally evaluated due to the small sample of included studies. Future trials should adopt longer follow-up and standardized reporting of PFS/OS, late adverse events, and relapse phenotypes. Further large-scale, randomized controlled studies or well-designed matched-cohort comparisons are also warranted to verify efficacy and safety, optimize CAR-T construct design and dosing, clarify patient stratification strategies (e.g., age, frailty, extramedullary disease, cytogenetic risk), and define the optimal clinical positioning of CD38-directed CAR-T therapy in RRMM treatment sequencing (35, 38, 62–64).

## 5 Conclusion

In summary, CD38-directed CAR-T cell therapy yields promising efficacy and a manageable safety profile in patients with RRMM, especially dual-target CD38/BCMA constructs that consistently elicit deep and molecular responses. However, single-target CD38 CAR-T remains limited by insufficient clinical evidence. Future investigations are warranted to conduct large-scale randomized controlled trials, optimize CAR-T structure design to improve response durability and reduce toxicities, and develop strategies to overcome antigen escape, with the ultimate goal of extending remission in RRMM.

## Data Availability

The original contributions presented in the study are included in the article/**Supplementary Material**. Further inquiries can be directed to the corresponding authors.
