# Supplementary material for: Efficacy and safety of CD38-directed CAR-T cell therapy for multiple myeloma: a systematic review and meta-analysis
Source: Front Oncol. 2026 Apr 29;16:1744250. doi: 10.3389/fonc.2026.1744250 (PMC13167427; doi:10.3389/fonc.2026.1744250)
Supplement: Supplementary file 1 [file DataSheet1.docx]

**
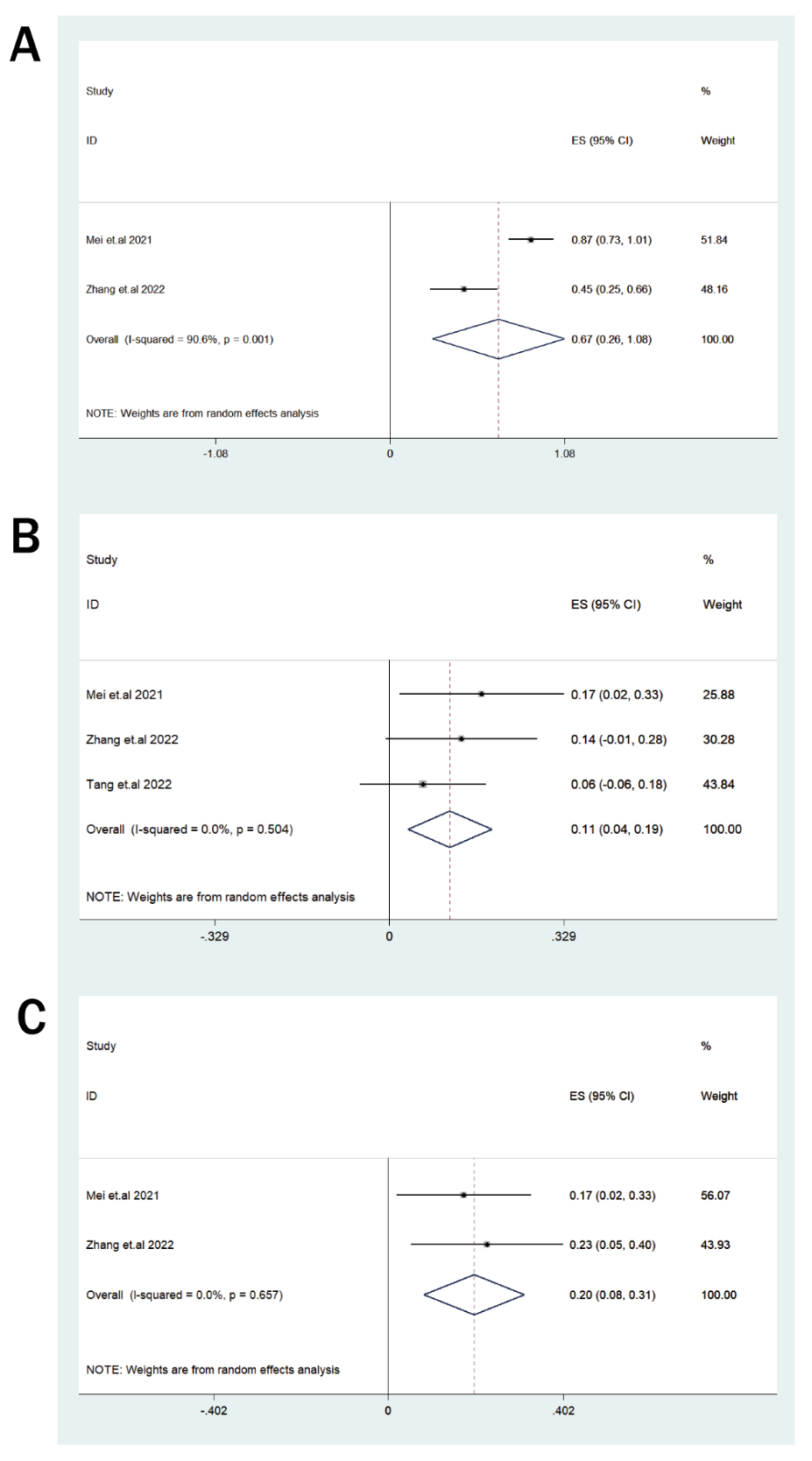
**

Fig. S1A–C. Forest plots for efficacy outcomes in patients with RRMM receiving CD38-directed CAR-T therapy: (A) MRD negativity, (B) PR, (C) VGPR.

**
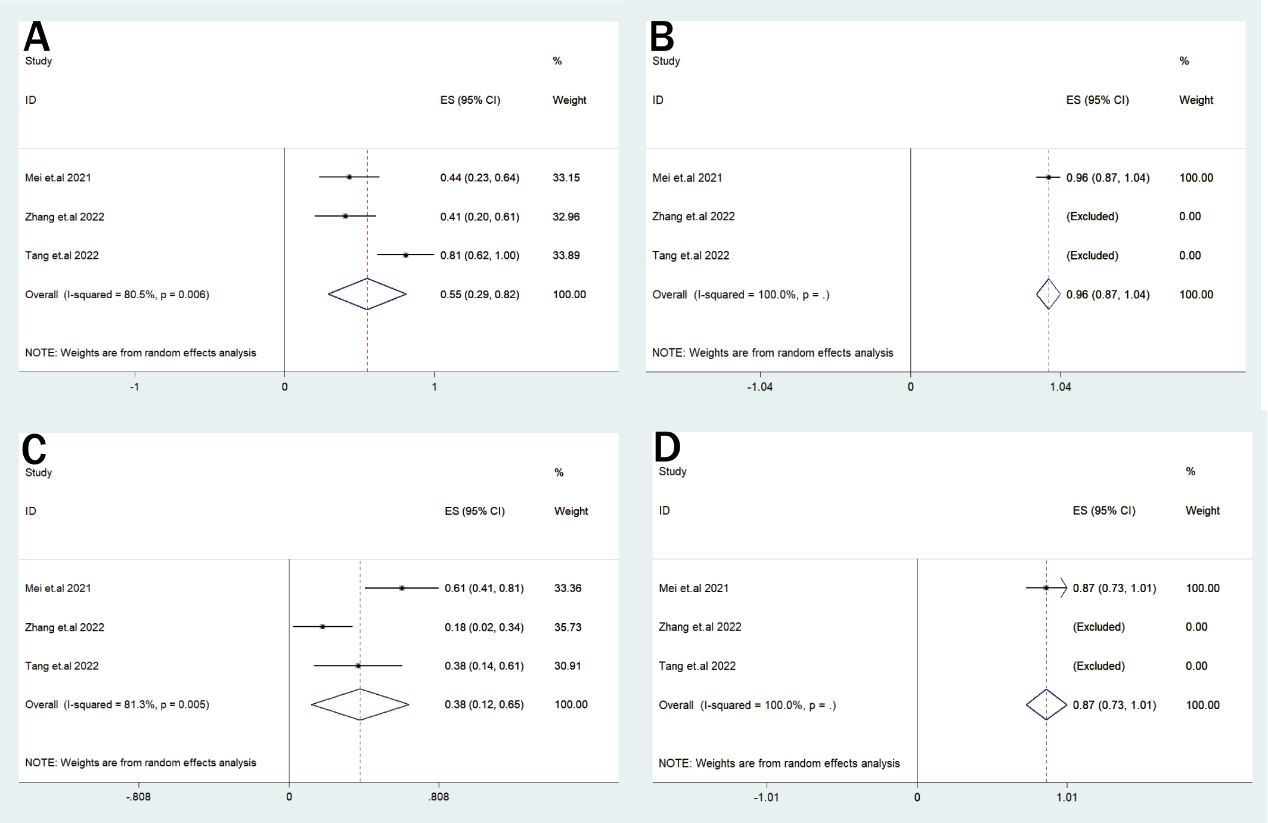
**

Fig. S2 A–D. Forest plots for common adverse events in patients with multiple myeloma (MM) receiving CD38-directed CAR-T therapy: (A) Anemia, (B) Hematologic toxicity, (C) Thrombocytopenia, (D) Leukopenia.


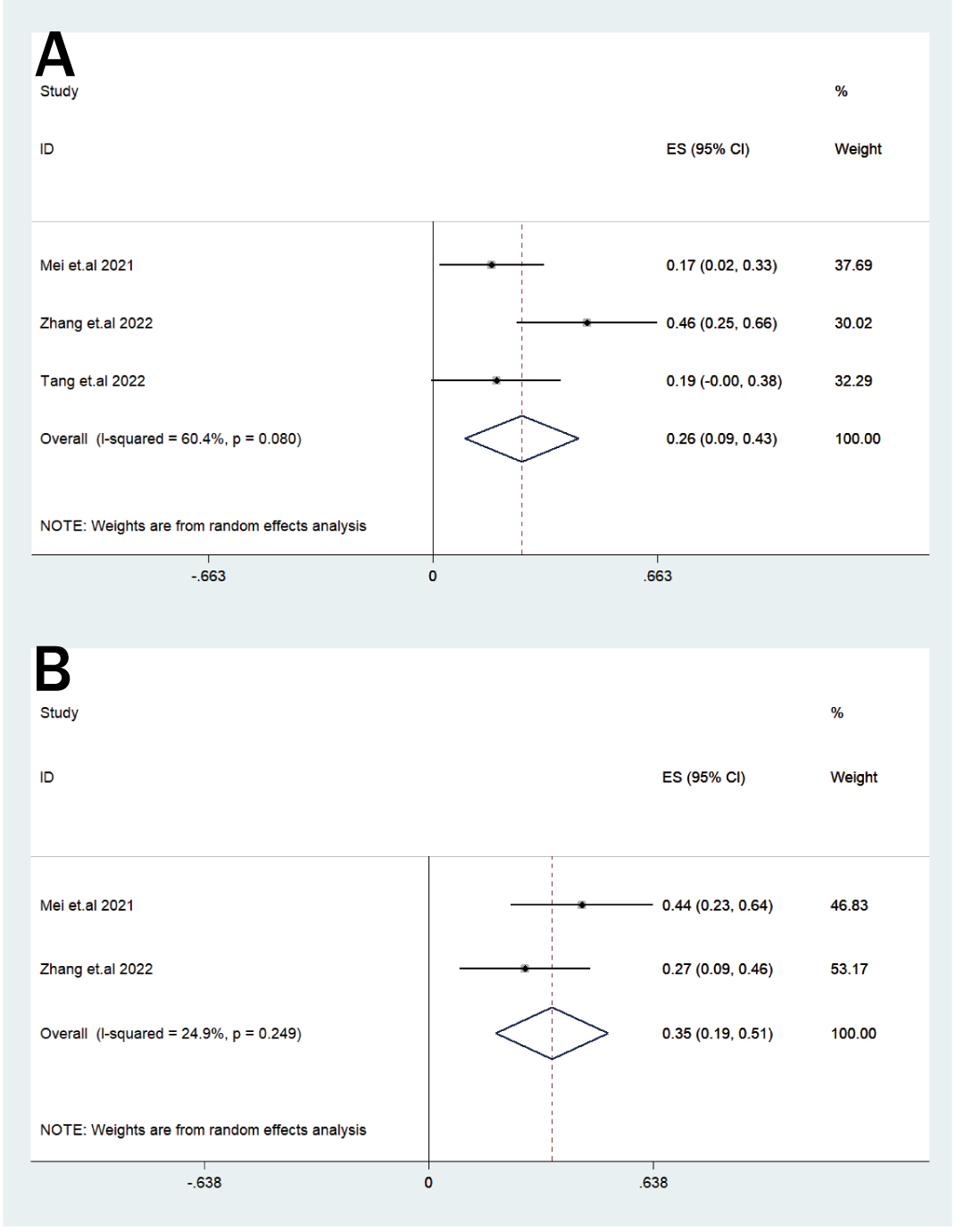


Fig. S3. Forest plots for other adverse events in patients with RRMM receiving CD38-directed CAR-T therapy: (A) Gastrointestinal reactions, (B) Impaired liver function.
